# Spinal anaesthesia to caesarean section: Patient satisfaction

**DOI:** 10.6026/973206300210459

**Published:** 2025-03-31

**Authors:** Tripti Vatsalya, Vikas Kumar Gupta, Rajni Thakur, Sonal Awasya

**Affiliations:** 1 Department of Anaesthesiology, Gandhi Medical College, Bhopal, India

**Keywords:** Spinal anaesthesia, maternal satisfaction, caesarean section

## Abstract

Patient satisfaction on spinal anaesthesia to caesarean section is of interest. Hence, data from 60 female doctors of various disciplines working in Bhopal city who underwent caesarean section under spinal anaesthesia during the last 5 years were included in this study. Factors associated with dissatisfaction were lack of communication and explanations for anaesthesia. Level of satisfaction with pain control was (86.6%). Pre-operative complications which scored highest are intraoperative shivering (10%), post-operative pain (20%) and backache (18.3%). Thus, the overall satisfaction among doctors receiving spinal anaesthesia for caesarean section was significantly high and major contributing factors for dissatisfaction need to be addressed.

## Background:

Spinal anaesthesia is the choice of anaesthesia for delivery via caesarean section in most of the health care centers in India. There are various studies focusing on specific time frames (initiation of analgesia) [1] characteristics of analgesia (sensory or motor block) [2], mode of delivery [3], or incidence of side-effects and complications, but the whole process of delivery and experience is also important. Patient satisfaction is an important measure of the quality of healthcare. Satisfaction with anaesthesia can provide feedback for performance and involvement and revalidation of agendas for healthcare professionals. Patient satisfaction represents their attitudes toward aspects of care as well as patients' emotions, feelings and their perception of delivered healthcare services [4, 5, 6-7]. Therefore, it is of interest to report on patient satisfaction on spinal anaesthesia to caesarean section.

## Materials and Methods:

This cross sectional survey was carried out on 60 female doctors of various disciplines working in Bhopal city who underwent caesarean section under spinal anaesthesia during the last 5 years. Doctors who received general anaesthesia and epidural anaesthesia were excluded from the study. A 12 item questionnaire (Annexure 1) was developed which included 5 sections to determine the satisfaction

[1] Preliminary information

[2] Communication and decision making

[3] Intraoperative events

[4] Post-operative care and occurrence of complications.

[5] Future preference of anaesthesia

The questionnaire was distributed to the study population. Data was analysed using SPSS version 17. The satisfaction scores are presented as a percentage.

## Results:

Total number of emergency cases = 31(51.6%)

Total number of elective cases =29 (48.3%)

As depicted in (Figure 1 - see PDF), the percentage of satisfaction in different domains as expressed by participants is as follows:

## Satisfaction with involvement in decision making during anesthesia:

Satisfied - 55 (91.6%)

Not satisfied - 5 (8.3%)

## Satisfied with communication and explanation given for anesthesia:

Satisfied - 46 (76.6%)

Not satisfied -14(23.3%)

## Level of satisfaction with pain control:

Satisfied - 52 (86.6%)

Not satisfied - 8 (13.3%)

## Initiation of breastfeeding:

Same day - 53(88.3%)

Next day - 7(11.6%)

## Overall satisfaction score:

Satisfied - 53 (88.3%)

Not satisfied - 7(11.6%)

## Future preference for spinal anaesthesia:

Yes - 52 (86.6 %)

No - 8 (13.33%)

## (Figure 2 - see PDF) expresses the contributory factors leading to anxiety among participants:

[1] Baby =50(83.3%)

[2] Anaesthesia = 4(6.6%)

[3] Surgery = 6(10%)

The major concern was related to the baby.

## Discussion:

Patient satisfaction is an important measure of the quality of healthcare and is used as an outcome measure in interventional and quality improvement studies. Its measurement is required to fulfil performance improvement and revalidation agenda for healthcare professionals [8]. Spinal anaesthesia remains the most common mode for caesarean section, due to advantages which include wakeful mother, minimal depression of newborn, post-operative analgesia and avoidance of risk of general anaesthesia [1]. For anaesthetic care providers, patient's satisfaction can be used to assess the actual impact of the procedure on the patients themselves. This can be assessed by a questionnaire which is more sensitive because the satisfaction tools in it involve various physiological and psychological aspects of patients. Here, we want to assess maternal satisfaction or problems after cesarean section under subarachnoid block, faced by medical personnel specially doctors. We have chosen doctors as our study population because being doctors they are well aware of the hospital environment and better know about every medical procedure. Thus, assessment of their satisfaction will definitely give us more reliable and valid data. This is very well proven by the 100% response rate of questions. Potential confounding bias like age, education, type, extent and duration of surgery were tried to be eradicated. Although confounding bias like the centre of surgery could not be prevented. It has been observed that the decision regarding the choice of anaesthesia is mostly suggested by the attending anesthesiologist (65.3%) and only 26.7 % of respondents were involved in decision making. It is very strange to know that (23.07 %) of doctor participants were not explained about the procedure. Participants in which surgery was on the basis of emergency had lower satisfactory score; lack of pre-anaesthetic visit, presence of labour pain, urgency of the surgery, busy schedule of an anesthesiologist or considering the knowledge of participants being a doctor are the contributing factors [9]. But this lack of communication between anesthesiologist and parturient can act as a negative predictor of maternal satisfaction. This has also been observed by studies of Porter *et al.* [10]. (11.4%) of participants were anxious regarding anesthesia and its complication; this can be attributed to poor communication which to some extent can be brought down by reassurance. Maheshwari *et al.* [3] in their study found a high level of anxiety among educated patients due to their awareness of complications. Yet some studies failed to show any correlation of anxiety with female sex [11]. Important factors causing dissatisfaction which were mentioned by the responders are shivering - post operative pain - nausea, vomiting, backache (Table 1). Kumar *et al.* [12] also noticed shivering as the most common side effect of subarachnoid block in parturient. A decrease in satisfaction score was observed in respondents due to post-operative backache reported by (31.4 %) of doctors. Backache is one of the complaints that can arise after spinal anesthesia, being one of the most common causes that make patients afraid to undergo spinal anesthesia and as many as 13.4% of the patients have backache as the major reason for refusing spinal anesthesia [13]. The cause of post-spinal backache is thought to be caused by inflammation at the injection site, back muscle spasm, and myalgia. Risk factors contributing to dissatisfaction were hospitals at which the anesthesia was administered and intraoperative pain. Fear of awareness, pain, several puncture attempts, and postural puncture headache were the main reasons for refusal to have spinal anesthesia again [14]. Initiation of breastfeeding has a strong influence on maternal satisfaction and all the responders were satisfied in this regard [15]. (86.6%) of respondents expressed their preference for receiving spinal anaesthesia in future which correlates with the finding of Makoko *et al.* [16].

## Conclusion:

The patient's opinion and decision regarding the mode of anaesthesia should be given priority and an anesthesiologist must duly explain about the procedure to be performed (spinal anaesthesia) before surgery. Anesthesiologists should have good rapport with their patients which will allay the anxiety of the patient and hence increase the satisfaction with anesthesia taking adequate measures to take care of factors like shivering, pain, backache which contribute to dissatisfaction. Thus, these factors should be considered to enhance the satisfaction rate of patients who are undergoing cesarean section under spinal anaesthesia.

## Figures and Tables

**Table 1:** Complications reported by participants (%)

**Intraoperative complications**	**Postoperative complications**	**Delayed complications**
Shivering - 10%	Pain = 20%	Backache = 18.3%
Vomiting - 3.3%	Headache = 5%	Numbness in lower limb - 1.6%
Partial effect - 3.3%	Backache= 1.6%	Hypertension = 1.6%
Hypotension - 5%	Vomiting = 6.6%	
	Shivering = 5%	
	Fever = 1.6%	
